# Improved Mutual Understanding for Human-Robot Collaboration: Combining Human-Aware Motion Planning with Haptic Feedback Devices for Communicating Planned Trajectory

**DOI:** 10.3390/s21113673

**Published:** 2021-05-25

**Authors:** Stefan Grushko, Aleš Vysocký, Petr Oščádal, Michal Vocetka, Petr Novák, Zdenko Bobovský

**Affiliations:** Department of Robotics, Faculty of Mechanical Engineering, VSB-TU Ostrava, 70800 Ostrava, Czech Republic; ales.vysocky@vsb.cz (A.V.); petr.oscadal@vsb.cz (P.O.); michal.vocetka@vsb.cz (M.V.); petr.novak@vsb.cz (P.N.); zdenko.bobovsky@vsb.cz (Z.B.)

**Keywords:** human robot collaboration, human robot interaction, path planning, bidirectional awareness, haptic feedback device, human machine interface

## Abstract

In a collaborative scenario, the communication between humans and robots is a fundamental aspect to achieve good efficiency and ergonomics in the task execution. A lot of research has been made related to enabling a robot system to understand and predict human behaviour, allowing the robot to adapt its motion to avoid collisions with human workers. Assuming the production task has a high degree of variability, the robot’s movements can be difficult to predict, leading to a feeling of anxiety in the worker when the robot changes its trajectory and approaches since the worker has no information about the planned movement of the robot. Additionally, without information about the robot’s movement, the human worker cannot effectively plan own activity without forcing the robot to constantly replan its movement. We propose a novel approach to communicating the robot’s intentions to a human worker. The improvement to the collaboration is presented by introducing haptic feedback devices, whose task is to notify the human worker about the currently planned robot’s trajectory and changes in its status. In order to verify the effectiveness of the developed human-machine interface in the conditions of a shared collaborative workspace, a user study was designed and conducted among 16 participants, whose objective was to accurately recognise the goal position of the robot during its movement. Data collected during the experiment included both objective and subjective parameters. Statistically significant results of the experiment indicated that all the participants could improve their task completion time by over 45% and generally were more subjectively satisfied when completing the task with equipped haptic feedback devices. The results also suggest the usefulness of the developed notification system since it improved users’ awareness about the motion plan of the robot.

## 1. Introduction

Human-robot collaboration (HRC) is a promising trend in the field of industrial and service robotics. Collaborative robots created new opportunities in the field of human-robot cooperation by enabling the robot to share the workspace with the personnel where it helps with non-ergonomic, repetitive, uncomfortable, or even dangerous operations. With the growing level of cooperation, there is a tendency to increase the intertwining of human and robot workspaces in the future, potentially leading to complete unification [1,2]. By allowing a fully shared workspace between humans and robots, it is possible to utilise the advantages of both, maximise their efficiency and minimise the time needed to complete a task. Shared workspaces are examples of a dynamic environment, as the human operators represent moving obstacles, which motions are difficult to predict accurately. Moreover, in a typically shared workplace, the collaborative robot does not have any perception about the position of the operator and can only react to the collisions by detecting the contacts with the tool or robot body (by measuring joint torques or monitoring the predicted joint position deviation [1,3]). These approaches have apparent limitations defined by the fact that in the case of adaptive tasks with high variability, the operator may be unaware of the actions planned by the robot, and the robot cannot predictively avoid a collision with the operator. In such adaptable HRC scenarios, the understanding between the human operator and the robot is crucial. On one side, the robot must be aware of a human operator, and on the other side, the operator needs to be aware of the current status of the robot system. Advanced workplaces may include monitoring systems [4,5,6] enabling the robot to react to (and potentially predict [7,8,9,10,11]) the operator’s movements by immediately stopping the activity or by replanning the trajectory [12,13,14]. However, a workspace monitoring system enabling the robot to avoid collisions can be considered as one-side awareness, but the other side of communication remains unresolved. An extensive review of the existing challenges in the field of human-robot interaction is available in the work of P. Tsarouchi et al. [15]. Despite many efforts made to make robots understand and predict human actions, there is still a lack of communication from the robot to the human operator. Difficulties in understanding the robot’s intent (planned trajectory, current task, internal status) during demanding cooperation can lead to dangerous situations, reduced work efficiency, and a general feeling of anxiety when working close to the robot (even if it is a collaborative robot). Better awareness can be achieved by providing the operator with intuitive communication channels that allow them to understand the motion plans and status of the robot. These communication channels may be realised with the help of feedback devices—Human-Machine Interfaces (HMIs). To convey information, these systems may utilise the primary sensory modalities of a human: vision, hearing, touch.

Many existing methods for communicating robot motion intent use graphical clues which notify the human worker about the status of the robot. Typical information for visualisation may include the internal status of the robot, command acknowledgement, planned trajectory, and current workspace borders. In the simplest example of such an approach, the data visualisations are displayed on 2D monitors [16] (static or hand-held tablets), which, however, require the operator to interrupt the current task and check the visualisation on display. Light projectors represent a straightforward solution for representing additional graphical clues and notifications to the operator, possibly directly in the operator’s vicinity, making it easier for the clues to be noticed [17,18]. Projector-based systems have a number of limitations, the main one being that various obstacles and the operator himself can block the system from both displaying the graphical clues and tracking the work objects, leading to an increased risk of misinterpretation of the visualised information by the operator.

Multiple Augmented Reality (AR) and Mixed Reality (MR) approaches have been developed as a subsequent improvement of the projector-based solutions. It allows a more intuitive overlay of the visual notifications with the real environment and objects in the workspace. Augmented reality headsets allow 3D graphics to be displayed directly in the user’s field of view without completely overlapping visual information from the real world [19,20,21]. One of the problems associated with the visualisation of the planned movement of the robot and other contextual information is that it cannot be guaranteed that this information is always in the field of view of the operator (the operator may be watching in another direction). It is also worth mentioning that MR/AR devices themselves present an interfering component that may distract the operator during the task. Experimental user studies performed by A. Hietanen [21] performed a comprehensive comparison and evaluation of HRC in a realistic manufacturing task in two conditions: a projector-mirror setup and a wearable AR headset. The results indicated that HoloLens was experienced less safe (comparing to the projector-based notification system) due to the intrusiveness of the device. Even though it was used as an augmented reality display, it blocked, to some extent, the view for the operator. An extensive review of the collaborative aspects of graphical interfaces for supporting workers during HRC is covered in the work of L. Wang et al. [2].

Another option of improving the awareness of a human worker during HRC is by utilising audio feedback. Auditory cues provide a wide range of contextual information that promotes awareness of a person about its surrounding. While vision feedback is traditionally preferred in applications that require a high level of accuracy, audio information is important in scenarios when other modalities are limited or blocked. An example of an application of this approach was demonstrated by A. Clair et al. [22], where the efficiency of the collaborative task was enhanced by enabling the robot to use synthesised speech to give a human teammate acoustic feedback about the currently performed action. G. Bolano et al. [14] focused on a multimodal feedback system for HRC by combining graphical and acoustic feedback channels. It is worth noting that due to potentially noisy manufacturing conditions, the operator may be unable to hear the audio signals.

Sense of touch represents a robust and direct way of transferring information to the user, making it suitable to convey information to workers in industrial environments, where visual and auditory modalities might be busy or blocked. P. Barros et al. [23] performed a set of tests using the simulation model of the teleoperated robot and enhancing the users with tactile feedback that could notify them about the actual collisions of the robot with the surroundings. Vibration devices can also be used during the control of an industrial robot, notifying the user about, for instance, approaching singularities and joint limits [24,25] or commencing the next phase of the manufacturing process [5].

It was demonstrated that the ability to communicate the robot’s motion to the worker in advance has an influence on the human propensity to accept the robot [2,26,27]. In this work, we propose a novel wearable haptic notification system for informing the human operator about the robot’s status, its currently planned trajectory, and the space that will be occupied by the robot during the movement. The haptic notification system consists of compact devices placed on both hands of the user, which provided vibrational alerts depending on the distance from the robot trajectory. Our approach combines the notifications with active collision avoidance [2], which enables the robot to continue on task execution even if the worker, despite the alerts, has precluded the initial trajectory.

Compared with existing interfaces, the proposed haptic notification system has the advantage of reliability alerting the user compared with graphical and acoustic feedback devices, whose efficiency can be limited or blocked during engagement in the task. The effectiveness of the robot’s trajectory intent communication to the user was verified in a user study. The results of the performed user study showed that the users had a better understanding of the robot’s motion. The developed haptic feedback system for collaborative workspaces can improve the efficiency and safety of human-robot cooperation in industrial conditions. The system may be able to reduce the time required for unskilled operators to get used to the manufacturing process and the movement of the robot in the near vicinity, along with helping to avoid the discomfort caused by unawareness about the intentions of the robot.

## 2. Materials and Methods

### 2.1. Concept

The proposed system is based on the concept of a shared collaborative workspace where the robot can adapt its movement to avoid collision with human workers. The workspace is monitored by multiple RGB-D sensors, and data provided by these sensors is used to construct a map of the robot’s surroundings and obstacles. At each step of the task execution, the robot creates a collision-free motion plan according to the currently available free space. If during the execution of the planned movement there is a change in the environment (for example, existing obstacles change their location) and the movement can no longer be completed due to possible collisions with obstacles, the robot can create a new motion plan (active collision avoidance). The improvement to the collaboration is presented by introducing haptic feedback devices (hereinafter, Human-Machine Interfaces, HMIs), whose task is to reliably notify the human worker about the currently planned robot’s trajectory and changes in its status. A wearable device is used to improve the operator’s awareness during the human-robot collaborative assembly task through vibrotactile feedback.

With regard to involvement in the work process, the hands are the parts of the body that are most often present in the shared workspace (especially when the work process is taking place at a table). For this reason, it was decided to develop a haptic HMI in the form of a compact device attached to the dorsal side of a work glove providing vibrational feedback to the user’s palm. Besides, it is a common practice in many industrial domains that the workers wear work gloves. Still, this placement requires a compact, wireless, and lightweight design that ensures the comfort of use (HMI must not limit the user’s capabilities during manual work). The human worker is equipped with two haptic feedback devices placed at each hand. The system utilises three types of notifications to inform the operator about the status of the robot: distance notification, replanning notification, and inaccessible goal notification.

**Figure 1:**
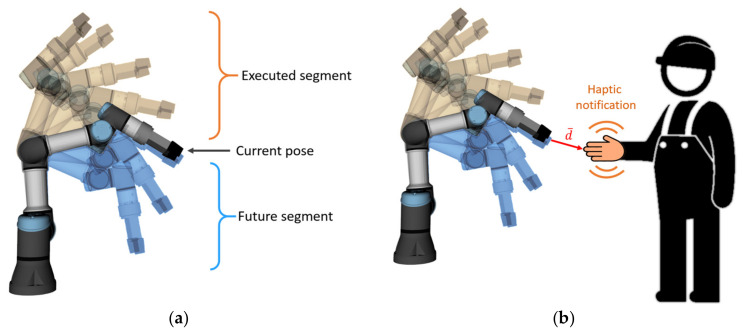
(**a**) Trajectory execution: already executed segment of trajectory, current state, and future segment (planned trajectory segment yet to be executed); (**b**) human worker equipped with haptic feedback device, which provides vibration alert about the proximity to the future trajectory of the robot; collision vector is denoted as $\vec{d}$. Note: collision vector is calculated considering all points of the robot body, including all links and joints.

The distance notification provides a continuous vibration alert to the user about the proximity to the currently planned trajectory of the robot (see Figure 1a). The future trajectory segment is defined as the part of the feasible trajectory that yet has not been executed (see Figure 1b).

**Figure 2:**
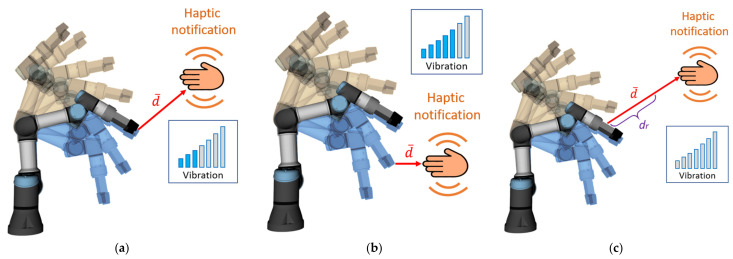
Illustration depicting the principle of distance notification: (**a**,**b**) vibration intensity is proportional to the distance to the closes point of the robot body in any timestep of the future trajectory; (**c**) distance notification is not active when the hand is further than the safe distance (reaction distance *d_r_*).

The closer the worker’s hand (equipped with HMI) approaches the future segment of the trajectory, the stronger the vibration provided by the device (see Figure 2a,b). The length of the vector between the nearest points of HMI and the robot body in all timesteps of the future trajectory (hereinafter “collision vector”, $\vec{d}$) is considered to be the distance, which is used to calculate the vibration intensity. Calculation of collision vector considers all the links and joints of the robot. The user receives vibration notifications while approaching any part of the robot body. There is also an upper limit of the distance at which HMIs provide feedback (reaction distance *d_r_*, Figure 2c). This ensures that the worker receives an alert only if his/her current actions may interfere with the robot trajectory.

The vibration intensity of the distance notification is calculated according to (1).

(1) $$v_{d}=\frac{(v_{p,max}-v_{p,min})*\left(d_{r}-\left|\vec{d}\right|\right)\;}{d_{r}+v_{p,min}}$$

where

$v_{d}$—calculated vibration intensity for distance notification,
$v_{p,max}$—maximum vibration intensity for distance notification,
$v_{p,min}$—minimum vibration intensity for distance notification,
$d_{r}$—reaction distance—distance threshold at which the distance notification is activated,
$\vec{d}$—current collision vector (see Figure 1).

The reaction distance $d_{r}$ was set to 15 cm as it was found the most convenient for the users. The calculated intensity $v_{d}$ is applied to all tactors of the corresponding haptic device.

**Figure 3:**
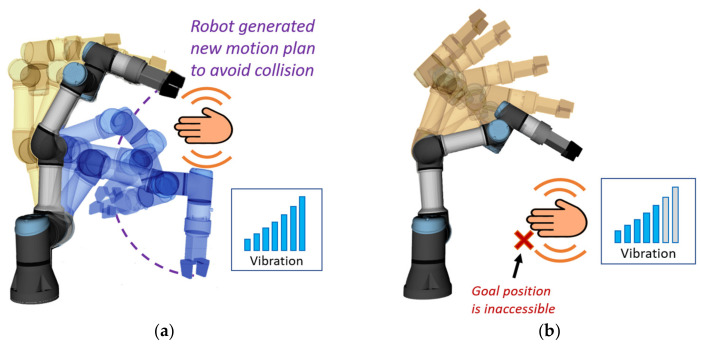
Status-related notifications: (**a**) replanning notification; (**b**) inaccessible goal notification.

When a person, despite a warning, interferes with the currently planned trajectory, the robot attempts to find a new feasible path to the goal position and continue the activity. Every time a new trajectory is planned, as a result of an environment change, both feedback devices use strong vibration notification (hereinafter “replanning notification”; this notification has a duration of 0.3 s) to draw the attention of the human worker and to indicate that the robot has detected an environment change and has replanned its movement (Figure 3a). If no feasible path to the target is found, both feedback devices also provide a strong vibration alert (hereinafter “inaccessible goal notification” Figure 3b), but this alert will last until the robot is able to continue its activity (movement to the goal). Both notifications may be utilised not only due to the obstacle presented by the hands of the user but also because of any obstacles (work tools, miscellaneous personal items) present in the workspace since the monitoring system maps all obstacles in the environment (using three depth sensors placed at the workplace). This way, even if the hands of the user will be completely hidden from the upper camera (which provides data for HMI localisation), the robot will be able to avoid collision with the user.

Vibration intensities were handpicked to make the haptic feedback unobtrusive when active. Vibration intensities for individual notifications are shown in Table 1, where the intensity of vibration is proportional to the provided PWM duty cycle represented in the range 0–100%: 0% represents no vibration, 100% represents the maximum attainable vibration. Preliminary tests have demonstrated that the vibration motors have high inertia and start spinning only at intensities above 30% PWM duty cycle. However, it was also noticed that for most users, only vibration above 50% of maximum intensity was noticeable.

**Figure 4:**
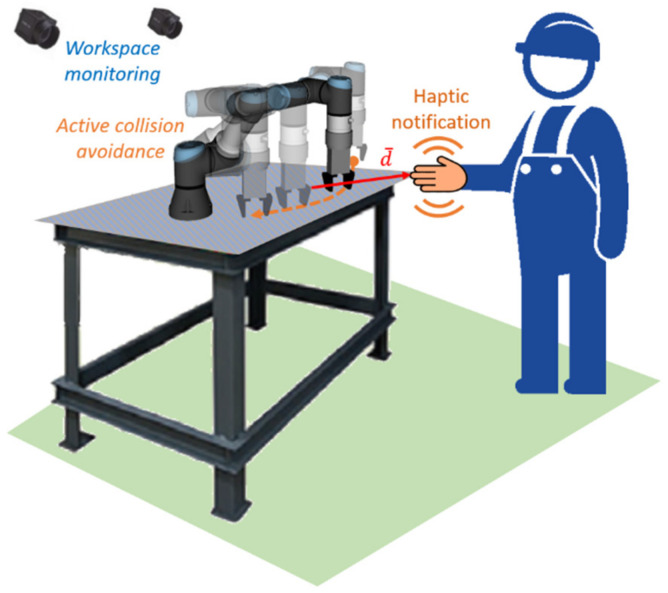
The concept of improved HRC combines principles of active collision avoidance with an increased awareness provided by the wearable haptic feedback device.

Taken together (see illustration in Figure 4), it is anticipated that the introduced system would minimise the interference between the robot and worker, leading to less frequent replanning, improve the comfort and acceptance for the human worker while working close to the robot and allow more fluent collaboration. The transparent behaviour of the robot can also lead to increased efficiency in performing the task since the worker will be informed when hindering the robot from continuing its activity.

### 2.2. Experimental Workspace

**Figure 5:**
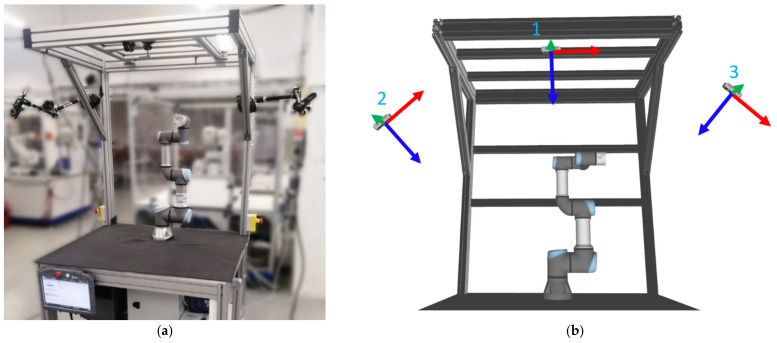
Experimental workspace: (**a**) overview; (**b**) Locations of three RGB-D sensors. View directions (*z*-axes) are depicted by the blue vectors.

The proposed system was tested on an experimental workspace with Universal Robots UR3e collaborative robot (Universal Robots, Odense, Denmark, see Figure 5a). UR3e robot has 6 DoFs, a working radius of 500 mm, and a maximum payload of 3 kg. In order to correctly map the obstacles within the workspace, three RealSense D435 RGB-D cameras (Santa Clara, CA, USA, see Figure 5b) are mounted on the workplace frame at different locations. Due to the principle of operation of the sensors, there is no limit on the number of cameras observing the same object simultaneously since the cameras do not interfere with each other. The streaming resolution was set to 424 × 240 at 15 FPS for both RGB and depth data streams, which is considered sufficient for the application.

**Figure 6:**
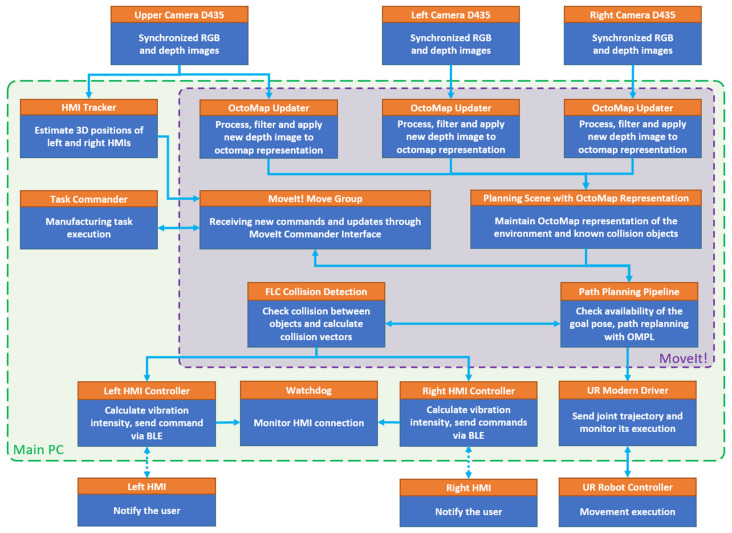
Data flow diagram.

It was decided to concentrate the control over the system into a single PC (Intel i7 2.80 GHz processor and 16 GB of RAM, Santa Clara, CA, USA) and to use the modular architecture offered by ROS (Melodic, Ubuntu 18.04, Linux, San Francisco, CA, USA) and to divide the software implementation into several separate components. The internal data flow is shown in Figure 6.

The task of HMI localisation is executed by the HMI Tracker node and will be explained in more detail further in the text. Task Commander node has the primary goal of abstracting high-level commands to the robot’s movement control and performing the production task. MoveIt! provides motion planning capabilities and calculations of collision vector during robot movement. Each HMI communicates with its software counterpart (HMI Controller) responsible for sustaining wireless Bluetooth data transfer, which is additionally monitored by a watchdog node. Communication with the real robot controller is implemented using a standard ROS driver.

**Figure 7:**
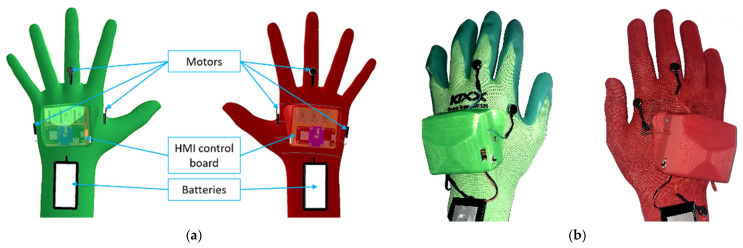
Prototype of HMI: (**a**) 3D model of HMI prototype, HMI cover is set as translucent in order to visualise the internal placement of the components; (**b**) implemented prototype.

The primary purpose of the developed haptic feedback devices is to provide the user with a reliable notification about the changes in the robot’s status and its currently planned trajectory. Both HMIs were implemented in the form of compact devices attached to the dorsal side of the working gloves (see Figure 7). This design ensures that the cover of the HMI control unit does not limit the user during manual work, as it does not restrict finger movements. The battery is placed on the user’s wrist, which also improves the overall ergonomics of the device. The total weight of a single HMI is 132 g. Each glove is equipped with six vibration motors, which provide haptic feedback. The motors are glued to the glove around the whole palm. The vibrational motors are controlled using DC motor drives (PWM control) by a single Arduino-compatible microcontroller that has an inbuilt battery management system and a Bluetooth Low Energy (BLE) chip, which enables communication with the main PC. During system operation, the PC constantly updates the vibration intensities of all HMI motors by sending the speed change requests via BLE. In the current version, the cover of the control unit is glued to the work gloves and cannot be detached.

It was decided to use distinctive colours for HMIs: the left HMI is green, and the right HMI is red. These colours will further simplify the task of hand tracking and determining the side of the hand.

### 2.3. Hand Tracking

The task of the HMI Tracker node is to determine the relative position of HMIs using data from the top depth camera located at the workplace. HMI segmentation from a 2D RGB camera image is simplified by the fact that the left and right HMIs have distinctive colours (it additionally greatly simplifies the correct determination of the side of the hand at different view angles). Colour-based segmentation is used as a simple alternative to the complex task of segmenting a 3D object in space, which otherwise would require the application of machine learning approaches such as SVM [28], deep learning-based object recognition [29], and image segmentation [30]. An extensive review of object localisation methods is demonstrated in the work of Y. Tang et al. [31].

The utilised colour-based HMI localisation approach has several limitations imposed by the nature of the detection process, the main one being that the arbitrary items with similar to the target (HMI) colours may interfere with localisation if present in the view. However, for our case, the localisation approach is considered sufficient, as it allows to reliably recognise the side of the hand regardless of the hand surface being partially occluded (providing that at least some part of the hand can be observed by the camera). In the future, the localisation system may integrate data from multiple cameras [13,32] available in the workplace in order to increase the stability and reliability of the tracking.

**Figure 8:**
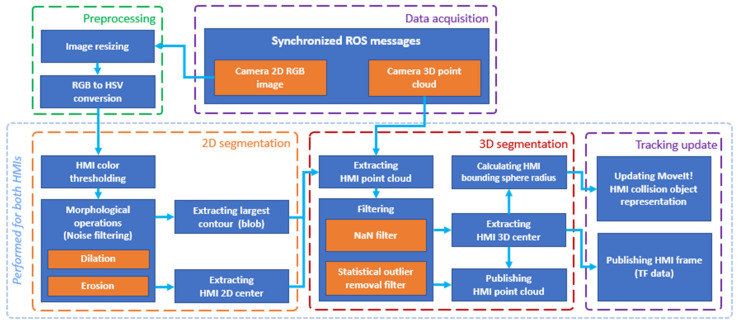
HMI Tracker data flow chart.

The position tracking of both HMIs is performed by an implemented ROS node called HMI Tracker. A schematic representation of the data processing at this node is displayed in Figure 8. Found HMI positions are published as transformations (ROS TF).

HMI Tracker node processes synchronised data messages from the upper depth camera ROS node. In order to mitigate the effect of environment luminance on the recognition accuracy, the 2D image from the camera is converted to the HVS colour space. The converted image is further processed by a 2D segmentation procedure (separately for each HMI). First, a colour range filter is used, and the resulting mask is further enhanced by the use of morphological operations (dilation and erosion), which allows for a reduction in noise in the computed mask. The largest contour is then located in the mask. The filtered HMI point cloud is then analysed to obtain the radius and the centre (centroid) of the HMI bounding sphere. The HMI bounding sphere is defined as the smallest sphere that envelopes all the HMI points, and its centre is defined as the geometrical centre of these points.

**Figure 9:**
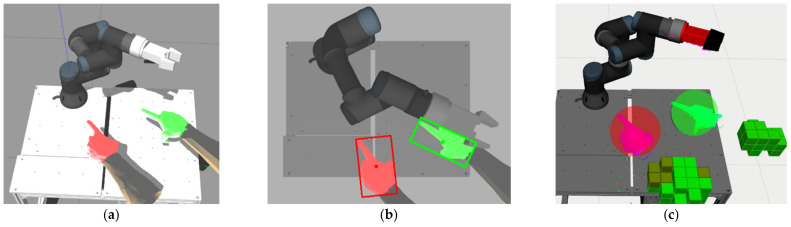
Snapshot of HMI tracking process: (**a**) Gazebo simulation; (**b**) upper camera image; (**c**) visualisation of the recognised HMI positions and segmented point cloud in RViz (HMIs are represented by spheres).

HMIs are internally represented as spheres, which allows a fast collision validation. A snapshot of the HMI tracking process (in the simulation model of the workspace) is shown in Figure 9.

### 2.4. Motion Planning

**Figure 10:**
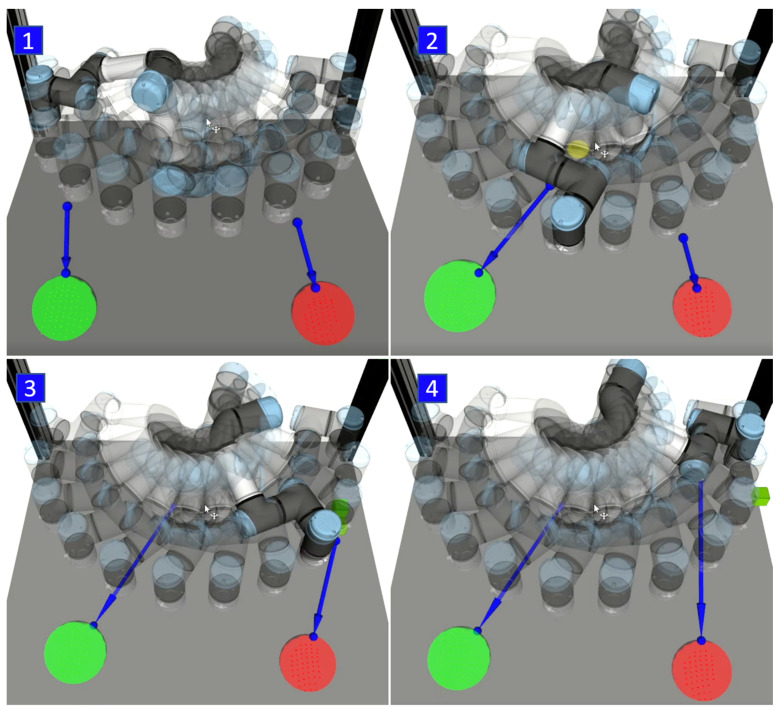
Example of how collision vector changing during motion sequence: robot moving from left to the right (**1**–**4**), collision vector is depicted by a blue arrow, HMIs are represented by green and red spheres.

The motion replanning subsystem is based on a modified version of ROS MoveIt! [33], which provides dynamic planning of the robot’s trajectory with respect to the current position of obstacles in surrounding space: if the operator precludes the currently planned movement of the robot, the robot is able to replan this movement to avoid collisions with the human operator and continue on the performed activity. However, it is assumed that the developed haptic feedback device will minimise the probability of interference between the robot and humans. Another task of MoveIt!, in addition to trajectory planning, is to calculate the distance between the HMI and the future trajectory of the robot. MoveIt! internally uses FCL (Fast Collision Library [34]) library to perform collision validation between scene object pairs. FCL also allows to perform a distance query that returns the nearest points between an object pair. We utilise this functionality to calculate the collision vector to both HMIs in each moment of the trajectory execution and publish it to other ROS nodes. An example of how calculated collision vector changes during the execution of a motion is illustrated in Figure 10.

## 3. Testing and User Study

Before starting the user studies, the system was tested in a simple task in both the real workspace and its simulated model (the parameters of the Gazebo simulation closely match those of the real workspace). During the testing, the task of the robot was to repeatedly move between two positions (from left to right and back, see Figure 10).

**Figure 11:**
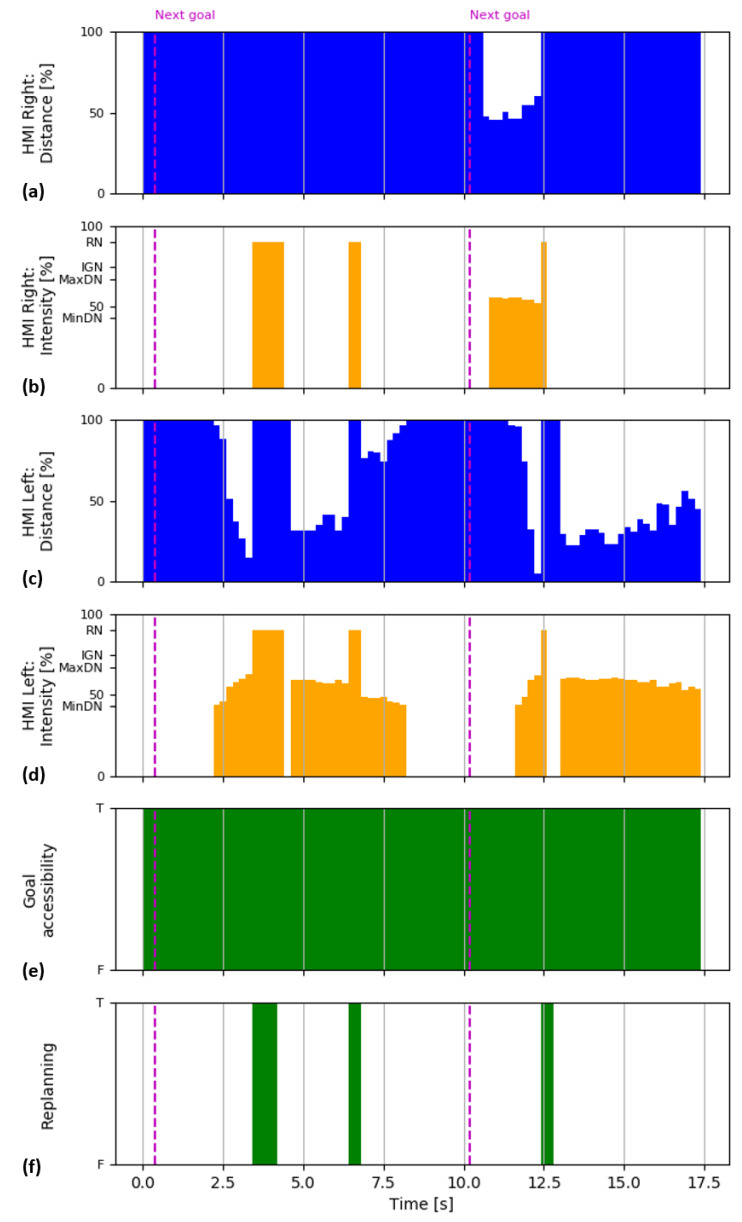
Timeline of the testing on the real workspace: (**a**,**c**) distances to HMIs displayed as a percentage of reaction distance; (**b**,**d**) momentary notification intensities displayed as a percentage from the maximum intensity, where RN—replanning notification, IGN—inaccessible goal notification, MaxDN—maximum distance notification, MinDN—minimum distance notification; (**e**) goal accessibility status where F—false, the planner was not able to find a feasible path to the goal); (**f**) status of replanning routine where T—true, path replanning was required; change of the goal is depicted by the purple dash line.

During the test, the individual system parameters were recorded and subsequently plotted as a timeline presented in Figure 11. The graph displays distances to both HMIs (magnitudes of the computed collision vectors), momentary notification intensities, goal accessibility status, and status of replanning routine.

Graphs in Figure 11 show that hands’ movement triggered trajectory replanning several times, causing activation of replanning notification. From the timeline, it can also be observed that notification intensity is in inverse proportion to the distance to HMIs. In general, it was observed that the reaction time of the system is mainly limited by the update rate of the collision object representation of the HMIs in MoveIt!: even though the HMI tracking rate is high enough to enable smooth processing at up to 50 Hz (currently intentionally limited to 15 Hz), the update time for MoveIt! collision objects are in a range from 7 to 10 Hz (which causes the step-like changes of the distance as shown in Figure 11). This set the limit to the overall reaction speed of the system, so fast movements of the user may remain undetected by the system; however, in the future, it can be addressed by distributing the process to multiple computation units.

### 3.1. User Study

**Figure 12:**
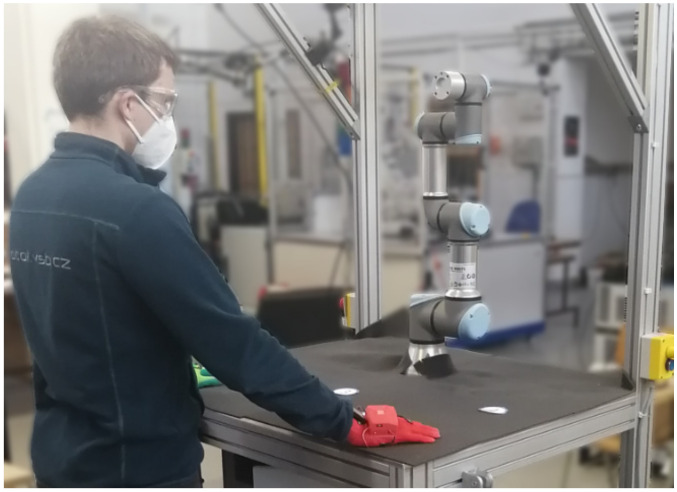
Performed user study.

In order to verify the effectiveness of the developed HMI (Human-Machine Interface) in the conditions of a shared workspace, a user study was developed (see Figure 12). The conducted user study included a testing system among 16 participants.

Data collected during the experiment included both objective (measured parameters of each testing cycle) and subjective (survey-based) parameters. Data analysis further allowed us to compare the interfaces and evaluate the research hypotheses.

### 3.2. Experiment Description

In order to evaluate the usability of the developed interface, the following experiment assessed the responses of the group of volunteers (test subjects, test participants), whose goal was to accurately recognise the goal position of the robot during its movement. The quantitative parameters measured during trials (time required to recognise the goal position and the percentage of the successfully recognised goals) were statistically evaluated as objective parameters in order to decide the status of the research hypotheses. Each volunteer additionally filled in the task-specific questionary consisting of questions related to the usability of each interface and the perceived naturalness during the task execution. The experiment was conducted with 16 volunteered university members, which included five unexperienced participants with the background different from the field of robotics. During the experiment run no harm was done to the volunteers.

**Figure 13:**
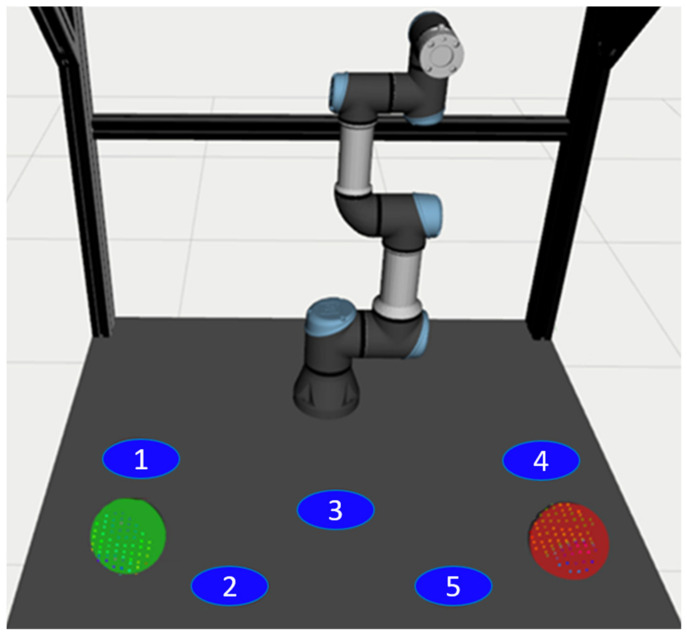
Preview of the workplace and the robot’s goal positions (marked with numbers 1–5); hand positions are marked with red (**right hand**) and green (**left hand**); the robot is in the initial position.

The experimental task was based on the idea of sorting parts into different containers, where it is not known in advance in which container the robot will have to place the next part. In each round of the experiment, the robot planned its trajectory from its starting position (this position did not change over the trials) to one of 5 possible TCP goal positions (see Figure 13). Each goal position was marked and numbered on the worktable.

During the experiment, each volunteer tested the following variations of completing the task:

- V1—Without HMI: the volunteer is not equipped with HMIs and has no feedback on approaching the robot’s future trajectory. The volunteer must determine the robot’s target position by visually examining the initial movement the robot makes to reach its goal.
- V2—Equipped HMIs: the volunteer is equipped with HMIs on both hands. The volunteer can use the HMI feedback (when moving hands) to determine the goal position of the robot. The volunteer is also instructed to watch their hands instead of watching the robot.

In both cases, the volunteers did not see a visualisation of the trajectory on the monitor.

The necessary safety precautions were taken during all the pilot experiments, and all the test subjects were informed about the potential risks and behaviour in safety-critical situations. Participation was not mandatory, and participants could leave any time they chose. All participants were required to review and sign a consent form and the definition of the experiment before beginning the experiment. After they agreed to participate, the experimenter clarified the ambiguities regarding the task and the principles of each interface. Additionally, before starting the experiment, each volunteer was allowed to observe the standard trajectory the robot takes while moving to all five predefined targets.

Each round started with a 3-2-1 countdown ensuring the volunteer knew the moment when the robots started to move. During movement to the goal position, the robot TCP speed was limited to the maximum value of 80 mm/s. The task of the volunteers was to determine the target position to which the robot is currently heading and, at the same time, avoid hindering the robot’s future path. The following results were possible:

- If the number reported by the volunteer was correct, the task completion time was recorded, and the attempt was counted as successful.
- In case that the goal position number stated by the volunteer was incorrect, the final result of the attempt was recorded as unsuccessful, and the measured time was discarded.
- If the volunteer intervened in the planned trajectory of the robot (or collided with the robot itself), the attempt was counted as unsuccessful, and the measured time was discarded.
- Each attempt was limited in time by the duration of the robot’s movement to the goal position. If the volunteer failed to determine the robot’s goal by this time, the attempt was counted as unsuccessful, and the measured time was discarded.

The objective parameters (time, percentage of success) of each attempt were measured and evaluated. At the end of the round, the robot automatically returned to the starting position at high speed (150 mm/s). If the user had reported the guessed goal position before the robot reached it, the person responsible for carrying out the experiment might have interrupted the movement of the robot and move it back to the initial position to spare the time of the experiment.

Testing of each interface option was performed in five rounds, i.e., a total of 10 rounds for each volunteer. Before testing a specific interface, the volunteer had three trial rounds. The order of the tested interfaces (V1, V2) was selected at random for each volunteer to mitigate the order effect on the measured parameters. In each round, the robot started its movement from the same starting position (arm vertically straightened above the worktable, see Figure 13), and the goal position of the robot was selected randomly (i.e., the randomly selected goal positions may have repeated multiple times). Before the start of each round, the volunteer placed hands on the starting positions marked in Figure 13; these positions were selected so that the user in the starting position did not preclude the robot in any of the target positions. At the end of all rounds, the volunteer filled in the questionnaire.

### 3.3. Hypotheses

The initial hypotheses are based on the suggestion is that the developed feedback system should improve the awareness and comfort of the human operator while working close to the robot. The transparent robot behaviour should also lead to an increase in efficiency in the completion of the task. The dependent measures (objective dependent variables) were defined as task completion time and task success rate. The within-subjects independent variable was defined as a robot intent-communication interface:

- V1: Without HMI—baseline.
- V2: Equipped HMIs.

Overall, it is expected that the test subjects will perform better (lower task completion times, higher task completion rate) and will have higher subjective ratings when equipped with HMIs. The experimental hypotheses were defined as follows:

**Hypothesis** **1.1.** **(H1.1):** *The efficiency of the test subjects will be greater with equipped HMIs than without HMIs.*

Higher efficiency is categorised as lower task completion time. This hypothesis is based on the suggestion that HMI contributes to task performance.

**Hypothesis** **1.2** **(H1.2):** *Time taken by each test subject to correctly determine the robot’s goal position will be similar during all rounds when equipped with HMIs (V2). In contrast, there will be high variation in task completion times between the rounds when the determination will be based solely on the available visual information.*

To test this hypothesis, task completion time will be measured for each subject in each task condition, and the standard deviation of the measurements will be compared. This hypothesis is based on the suggestion that users’ awareness enhanced by the haptic feedback is not influenced by the differences in the trajectories, whereas in the case of visual feedback, the awareness is dependent on the actual trajectory shape.

**Hypothesis** **2** **(H2):** *Volunteers will subjectively percept the task as simpler when performing the tasks while equipped with HMIs (V2) than by relying solely on the available visual information (V1).*

This hypothesis is based on the suggestion that the haptic feedback significantly contributes to the user awareness about the future trajectory of the robot, thus making the task cognitively easier.

Efficiency was defined as the time taken for the human subjects to complete the task and effectiveness as the percentage of successful task completion. Efficiency and effectiveness were evaluated objectively by measuring these parameters for each test subject during rounds of the experiment.

Apart from objective parameters, multiple subjective aspects of interacting with different types of interfaces were mapped. The analysis of subjective findings was based on responses to 17 questions. The main points of interest focused on the understanding of the robot’s goals and motions, the feel of security when working closely with the robot, ergonomics, and the overall task difficulty. For both collaboration approaches, the participants were asked to indicate on a 1–7 Likert scale (scaling from 1—“totally disagree” to 5—“totally agree”) the extent to which they agreed with the defined statements. Questionnaire items were inspired by works by R. Ganesan et al. [35], G. Bolano et al. [14], and A. Hietanen [21]; however, due to the differences in the tested interfaces, the questions have been significantly changed.

The first four questions (Q1–Q4, see Table 2) were task- and awareness-related and aimed to map the comparative aspects of the collaboration during the task execution with all tested interface variants V1, V2 and to test the general clarity of the provided instructions.

For HMI interface (V2) were additionally stated the questions defined in Table 3.

The test subjects also could choose an interface of their own preference. The volunteer could additionally leave a free comment about any topic related to each interface option. To minimise the effect of bias (practice effect [36]) caused by the order in which participants interacted with each interface, the order was chosen randomly for each participant during the experiment.

## 4. Results

To determine whether the differences between objective measures in the conditions (V1, V2) were significant at the 95% confidence level, *t*-test was applied.

**Figure 14:**
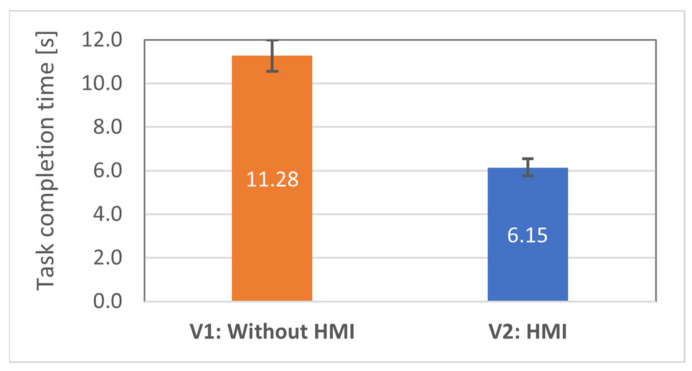
Average task completion time with standard errors for all 16 participants: lower is better.

Hypothesis H1.1 states that the efficiency of the human-robot collaborative team will be higher in the case of the equipped HMIs (V2). The total time taken for completing the tasks was measured and compared between the conditions. *t*-tests revealed statistically significant difference (average improvement over 45%) in mean task completion times between V1 (M = 11.28, SE = 0.71) and V2 (M = 6.15, SE = 0.38) interfaces, *t*(15) = 6.34, *p* < 0.00001, Figure 14. Thus, hypothesis H1.1 was supported.

**Figure 15:**
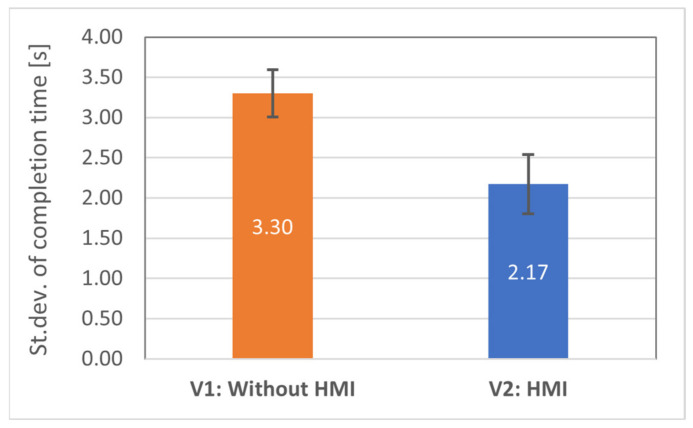
Standard deviations of the task completion time: lower is better.

Hypothesis H1.2 stated that the time taken by test subjects to correctly determine the goal position would be similar in all rounds when equipped with HMIs (V2). To investigate the hypothesis, the task completion time for all test subjects was analysed using the standard deviations. It was observed that the standard deviation for each subject for HMI modes (M = 2.17, SE = 0.37) was significantly lower than for visual inspection (V1: M = 3.30, SE = 0.29), implying that V2 allowed the participants to perform the task within a similar amount of time, whereas the time need with V1 interface was highly distinct in each round—see Figure 15. The deviations were compared using *t*-test, and statistically significant differences were found, *t*(15) = 2.27, *p* < 0.019. This suggests that the provided haptic feedback was intuitive and took approximately the same amount of time for the participants in each round to determine the goal position, thus supporting the H1.2 hypothesis.

**Figure 16:**
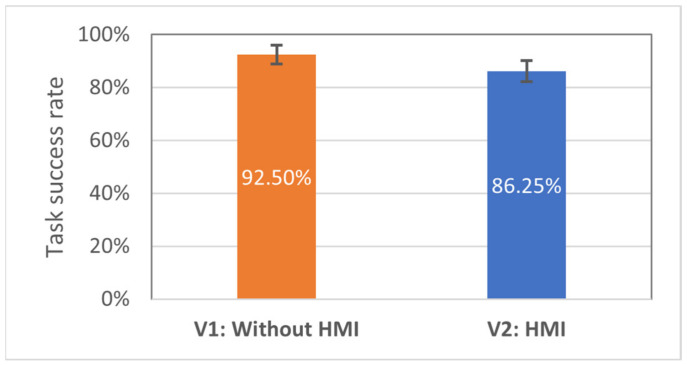
Average task success rate for all 16 participants: higher is better.

It is also anticipated that the effectiveness of the V2 and the baseline will be comparable; however, proving this hypothesis (basically null-hypothesis) with a high confidence level requires a large number of test subjects. The average task success rates with standard errors are shown in Figure 16. One of the factors that may have led to a lower success rate with the V2 interface may be that the haptic feedback (V2) did not provide the test subjects with enough spatial information, which led to an ambiguous determination of the goal positions.

**Figure 17:**
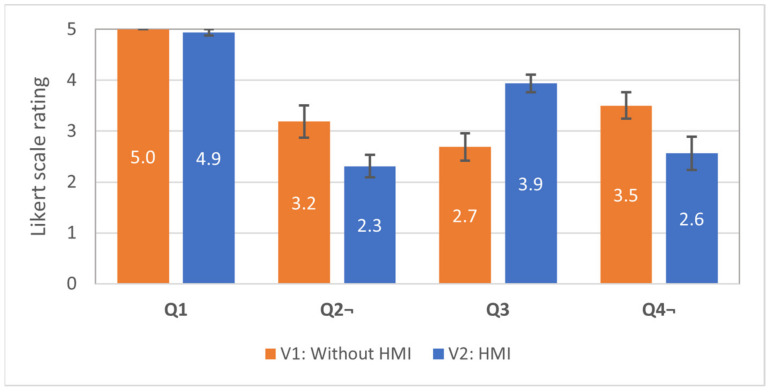
Average scores with standard errors for the questions Q1–Q4 used in the user studies (16 participants). Score 5 denotes “totally agree” and 1—“totally disagree”. Questions Q1, Q3—higher is better; Q2, Q4—lower is better.

All 16 participants answered a total of 17 template questions, and the results were analysed. The first four questions (Q1–Q4) were common for both interfaces and intended to capture the differences in subjective usability between them. The average scores with the standard errors are shown in Figure 17. To determine whether the differences between ratings for each questionnaire item between test conditions (V1, V2) were significant at the 95% confidence level, *t*-test was applied.

Q1 was intended as an indicator of clarity of the task between the test subjects. The results show that the test subjects understood the provided instructions (there are no statistically significant differences in the responses in all three conditions). Q2 was related to the subjective perception of the task difficulty in each condition. The *t*-test revealed a statistically significant difference in responses between V1 (M = 3.19, SE = 0.32) and V2 (M = 2.31, SE = 0.22) interfaces, *t*(15) = 2.67, *p* < 0.01, the test subjects perceived the task as more simple while equipped with HMIs. Q3 intended to compare the subjectively perceived improvement in the awareness related to the robot’s trajectory. *t*-test revealed a statistically significant difference in responses between V1 (M = 2.69, SE = 0.27) and V2 (M = 3.94, SE = 0.17) interfaces, *t*(15) = 3.873, *p* < 0.001, the participants subjectively perceived an improvement of their awareness about the robot trajectory when equipped with HMI. Q4 was designed to find out if the subjects subjectively felt the need for additional information in order to reliably recognise the trajectory of the robot. Statistically significant difference in responses was found between V1 (M = 3.50, SE = 0.26) and V2 (M = 2.56, SE = 0.33) interfaces, *t*(15) = 3.34, *p* < 0.01. Thus, hypothesis H2 was supported by the responses to Q2–Q3. The subjective findings are also supported by the previous evaluation of the objective performance of the test subjects.

**Figure 18:**
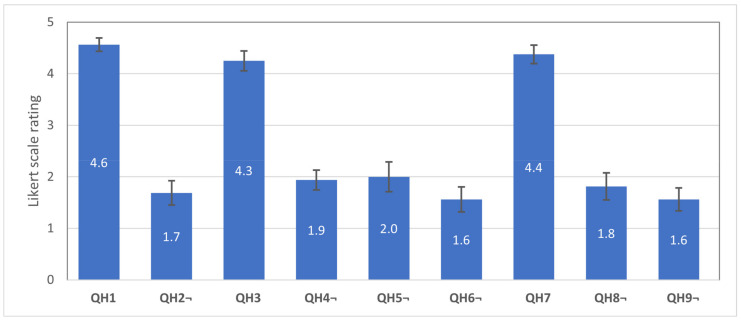
Average scores with standard errors for the questions QH1–QH9 used for evaluation of the HMI usability. Score 5 denotes “totally agree”, and 1—“totally disagree”. Questions QH1, QH3, QH7—higher is better; QH2, QH4, QH5, QH6, QH8, QH9—lower is better.

The additional QH1–QH9 questions were intended to capture the subjective usability of the HMI (V2) interface. According to the high ranking (see Figure 18), it can be concluded that the users were satisfied with the interface and the provided feedback. There were few participants who reported that the vibration feedback was too strong and participants who reported feedback as too weak, thus leading to a conclusion that the optimal intensity of vibration feedback should be further investigated or should allow personal adjustments.

The users could also choose the one interface of their personal preference: all users favoured the HMI interface (V2). The user survey additionally offered to leave a free comment for each of the tested interfaces. Major themes mentioned in the comments included user perceptions of the haptic feedback. Some subjects noted that the vibration intensity was perceived differently in different places around the arm (for example, the vibration of the tractor +Z was perceived less). This suggests that additional research is needed to find the optimal arrangement of vibration motors. Participants also noted that it was hard for them to recognise the movement of the robot to certain positions, since, for example, in the case of goal 1 and 2, there sometimes was an ambiguity in the provided feedback, which lead to incorrectly reported number (which manifested in the task success rate). Multiple test subjects mentioned that the gloves’ size was not optimal for their hands. This issue may be solved by implementing HMI in the form of modules that will be locked onto the universal work gloves.

## 5. Discussion

Evaluation of the objective parameters has shown significant evidence supporting hypotheses H1.1, H1.2. The participants took less time to recognise the goal position when equipped with HMIs. The time required by test subjects to successfully complete the task with HMIs equipped had low variation (unlike with visual feedback), potentially indicating that user awareness was independent of the shape of the robot movement.

Subjective findings from structured and free response questions supported hypothesis 2, which stated that participants would be more satisfied with the HMI interface compared to baseline. Overall, participants favoured the HMI with regard to human-robot fluency, clarity, and feedback.

Taken together, the results of the experiments indicate the usefulness of the developed system since it improves user’s awareness about the motion plan of the robot. While not conclusive, these results indicate a potential of a haptic feedback-based approach in improving the interaction quality in human-robot collaboration.

In order to assess the developed system, we critically analyse its features and limitations. To the best of our knowledge, the presented system is the first implementation of haptic notifications, which provide the user with the information about the space that will be occupied by the robot during the movement execution while simultaneously allowing the robot to adapt the trajectory to avoid collisions with the worker. Previous studies have only demonstrated utilisation of the haptic feedback for informing the user about robot reaching some predefined positions [5,23]. The chosen modality (haptic feedback) has the advantage of more reliable data transfer compared to the graphical and acoustic feedback, whose efficiency can be limited or blocked during the engagement of the worker in the task. The utilisation of the haptic feedback as an information channel may also off-load the corresponding senses (vision and hearing). Yet, it is worth noting that in certain cases, the executed task activity (such as handling an electric screwdriver) may block the perception of the provided vibration alerts in the worker’s hands. According to the results of the analysis of the subjective rating of the system, more investigation is needed in order to find the optimal placement of the vibration motors and vibration intensity levels, which will be considered equally sufficient by the majority of the users. This is necessary given that tactile sensitivity varies from person to person. However, this was beyond the scope of this study.

The HMI localisation system currently relies on data from a single depth camera, potentially enabling situations where the HMI would be occluded by an obstacle, leading to incorrect localisation. This, however, can be solved in the future by integrating data from multiple cameras into the HMI localisation pipeline. It should be emphasised that even if the hands of the user will be completely hidden from the upper camera, the robot will be able to avoid collision with the user since the obstacles in the workspace are mapped by all three cameras observing it from different perspectives. Since the overall reaction speed of the system is limited by 10 Hz updates of the workspace representation, swift movements of the user may remain undetected by the system; however, in the future, it can be addressed by distributing the process to multiple computation units and further optimising the system performance.

During the test, it was noted that there is a possibility to further improve the notification devices by enabling proportional activation of the tactors depending on the direction to the closest point of the robot’s trajectory and HMI’s orientation. This would allow avoiding ambiguity in the determination of the goal positions by providing the user with additional information. A similar solution was implemented in the work of M. Aggravi et al. [37], where they implemented a solution for guiding the hand of a human user using a vibrotactile haptic device placed on the user’s forearm. Another example was implemented in the study of S. Scheggi et al. [38,39], in which a mobile robot had the task of steering a human (possibly sightless) from an initial to the desired target position through a cluttered corridor by only interacting with the human via HMI bracelet with three embedded vibration motors.

## 6. Conclusions

In this work, we propose an approach to improve human-robot collaboration. The system is based on the concept of a shared collaborative workspace where the robot can adapt its movement to avoid collision with human workers. The workspace is monitored by multiple RGB-D sensors, and data provided by these sensors allow the construction of a map of the robot’s surroundings and obstacles. At each step of the task execution, the robot creates a collision-free motion plan according to the currently available free space. If during the execution of the planned movement there is a change in the environment and the movement can no longer be completed due to possible collisions with obstacles, the robot can create a new motion plan. The improvement of the collaboration is presented by introducing haptic feedback devices, whose task is to notify the human worker about the currently planned robot’s trajectory and changes in its status. The human worker is equipped with two haptic feedback devices placed at each hand. These feedback devices provide continuous vibration alerts to the user about the proximity to the currently planned trajectory of the robot: the closer the worker’s hand (equipped with the feedback device) approaches the future segment of the trajectory, the stronger the vibration provided by the device. A prototype of the proposed notification system was implemented and tested on an experimental collaborative workspace.

An experimental study was performed to evaluate the effect of the developed haptic feedback system for improving human awareness during HRC. The experiment data analysis included both quantitative (objective and subjective) and qualitative (subjective) findings from the experiment. Haptic HMI was found superior to the baseline and allowed the test subjects to complete the tasks faster.

Future research will focus on the improvement of the developed notification system to allow more information to be transferred to the user. The described concept of the haptic distance notifications may communicate not only the distance to the robot’s trajectory but also the direction of the nearest point of the trajectory (relatively to the corresponding HMI). By organising the vibration motors into a spatial structure resembling an orthogonal coordinate system, the vibration of each motor may alert the user with the direction of the possible impact. This improved approach additionally requires measuring the relative orientation of each HMI, which can be implemented by incorporating IMU sensors into the design of HMIs. The HMI localisation system may be further improved by combining and processing the data from all the available cameras in the workplace, which would ensure that the HMI can be localised even if it is occluded or is outside the field of view of multiple cameras as long as it is visible to one of them. More types of notifications may represent more states of the robot and the surrounding machines, allowing the user to quickly react in a situation when human intervention is needed. However, due to the computational and overall complexity of the system and the supporting device infrastructure, industrial implementation of the system would require a standalone processing unit in addition to the robot controller. In general, the usability of the system can be extended for use in large production line conditions, including several robots and automatic production systems. As a human worker moves along the production line to various machines and multiple robots, the system can provide notifications of any danger that is at a reaction distance from the human.

## Figures and Tables

**Table 1:** Notification vibration intensities.

Notification	Vibration Intensity (PWM Duty Cycle)	Duration	Description
Inaccessible goal notification	85%	Continuous	The robot was not able to find a feasible path to the goal
Replanning notification	95%	0.3 s	The robot has replanned its motion in order to avoid a collision
Distance notification (maximum)	80%	Continuous	The user’s hand is about to block the currently planned robot trajectory
Distance notification (minimum)	60%	Continuous	The user’s hand is approaching the future segment of the robot’s trajectory

**Table 2:** General questions for all the tested interfaces.

General Questions
Q1. The task was clear for me
Q2. The task was demanding
Q3. It was simple to determine the goal position of the robot
Q4. More information was needed to accurately determine goal position of the robot

**Table 3:** HMI-related questions.

HMI-Related Questions
QH1. HMI improved my awareness of the robot’s future trajectory
QH2. Work with HMI required long training
QH3. Work with HMI improved my confidence in safety during the task
QH4. Haptic feedback (vibration) from HMI was misleading
QH5. Haptic feedback (vibration) from HMI was too strong
QH6. Haptic feedback (vibration) from HMI was too weak
QH7. Haptic feedback (vibration) from HMI was sufficient
QH8. Haptic feedback (vibration) from HMI overwhelmed my perceptions
QH9. Use of HMI was inconvenient or caused unpleasant sensations during activation

## Data Availability

The data presented in this study are available on request from the corresponding author. The data are not publicly available due to project restrictions.
